# Annals of Medico-Psychology
*Annales Medico-Psychologiques. Journal destiné à recueillir tous les documents relatifs à l'Aliénation Mentale, aux Neuroses, et à la Médicine légale des Aliénés; par MM. les Docteurs Baillarger et Cerise. Octobre, 1849. 8vo, pp. 636. Paris.


**Published:** 1850-04-01

**Authors:** 


### Art. VI.—ANNALS OF MEDICO-PSYCHOLOGY.*

We purpose bringing before our readers a brief analysis of the contents of the last number of this journal, *seriatim*, and to point out the most interesting and valuable facts it contains, so far as our space will permit.

### I. *On the influence of Erysipelas of the Face and Scalp on the production of General Paralysis.* By M. Baillarger.

"If there be any one fact well established in the history of general paralysis," observes M. Baillarger, "it is assuredly the influence of cerebral congestion in the production of this disease. That in carefully studying its etiology, it is observed that its causes are efficient by the production of cerebral congestions; such, in the first rank, are the suppression of sanguineous discharges, excess in drinking, venereal excesses, moral excitement in subjects of a plethoric habit, epilepsy, &c. Among the causes also which may dispose to considerable afflux of blood to the brain, there is one that has been passed over almost in silence, and which, although less frequent than those

* Annales Medico-Psychologiques. Journal destiné à recueillir tous les documents relatifs à l'Aliénation Mentale, aux Neuroses, et à la Médicine légale des Aliénés; par MM. les Docteurs Baillarger et Cerise. Octobre, 1849. 8vo, pp. 636. Paris.

which have been mentioned, does not the less deserve attention, that is, erysipelas of the face and scalp."

M. Baillarger relates three cases which possess interest in this point of view. In the first case, five attacks of erysipelas of the head occurred, within the period of three years, in a woman predisposed to cerebral congestion. In the two subsequent instances, severe headaches became persistent after erysipelas of the head and face. In one of these, cerebral congestion and ambitious delirium supervened. In the other, the general paralysis occurred after excess in drinking. In both, the commencement of the disease could be distinctly dated from the attacks of erysipelas.

The point of practical importance in all is, that the persistent headaches should be regarded as indications of a very serious disease, and treated accordingly.

II. *On the Feeding of the Insane; the difficulties which it presents and on the means of obviating the inconveniences of forced alimentation.* By Dr. ANDREA VERGA. Translated from the Italian by Dr. L. LUNIER.

Dr. Verga notices the several forms of insanity in which the refusal of food is a prominent feature; and the motives which give rise to this refusal, as well as various manœuvres which have been successfully adopted to overcome this aversion. Among the latter he cites at length the plan adopted in an otherwise quite intractable case, by Dr. Filipi, of Milan. This was the introduction of two needles into the digastric muscles, and the connecting these with a voltaic pile of eight pairs. The mouth was opened widely, and the patient took his food. The plan was tried a second and a third time, and when the patient thus found that he could not resist a power which he came to regard as supernatural, it was sufficient to threaten him with the operation to induce him docilely to take his food; and thus he was cured, who would otherwise have died from inanition. But, as Dr. Verga observes, this plan is not so simple and easy in practice as it appears. The whole object is not gained when food is placed on the tongue, the difficulty is to get it swallowed; it is often rejected from the pharynx, even when introduced there by the help of instruments.

Dr. Verga sums up the reasons of his aversion to "forced alimentation" in the following proposition:—

1st. That it is difficult to know the motives which give rise to the refusal of food by the insane. In illustration of this objection Dr. Verga cites two cases reported by M. Baillarger. In one of these, forced alimentation was had recourse to, but in which case the patient died with diarrhœa and marasmus; and in whom the autopsy disclosed congestion of the right, and hepatization of the lower lobe of the left lung. In the second, forced alimentation was had recourse to during a hundred and seventy days. The patient died, and after death two ulcers of the stomach, hypertrophy of the heart, and hepatization of the right lung, were found. The same writer also records a similar case in which cancer of the uterus was discovered after death. In all these instances, Dr. Verga considers it most probable that the bodily disease was the cause of the refusal of food rather than the mental disorder. In all cases where the patients have died marasmic, under forced alimentation, Dr. Verga states that he has always found serious organic lesions. Whenever, therefore, he cannot positively detect the cause of the refusal of food, Dr. Verga observes, that he chooses the less evil, in not exposing the patient to an aggravation of organic lesions by the excitement of forced alimentation.

2nd. Since we are too often completely in ignorance of the cause of the refusal of food, this circumstance should ordinarily be regarded as indicative of some particular morbid state of the organism, or, at least, that the want of food is absent. Upon this principle, Dr. Verga explains the occurrence of cases of prolonged abstinence, in which, sometimes, no food is taken for weeks, or a very small quantity, indeed, for several years. The refusal of food must be considered as a rare symptom of organic disease. And it must be borne in mind, Dr. Verga observes, that these patients are nourished, not by what food they can be made to swallow, but by what they can really digest.

Dr. Verga alludes to those gastric and general derangements in which loss of appetite is a prominent, but temporary, symptom, and inquires what would be the result of forced alimentation in such cases, which he regards as differing only in degree and duration from the cases now under consideration.

3rd. The sight of the preparations to put in force the compulsory alimentation will often suffice of itself to overcome the refusal to take food. The repugnance to food will, in many cases, subside, if the patient be left to himself.

From the consideration of the entire subject, Dr. Verga comes to the conclusion that forced alimentation can only be admissible in the two following cases:—

1st. Where there is a manifest determination to perpetrate suicide by starvation, and the patient rebels against every other reasonable measure. In these cases, in Dr. Verga's opinion, M. Baillarger's tube is preferable to every other instrument.

2nd. In idiocy, with suspension of the voluntary movements of mastication and deglutition, and in which there exists only passive resistance.

In all other cases, Dr. Verga considers that the refusal may be overcome by prudently waiting, and using gentle persuasion, with other general means. And where the refusal of food is traced to some morbid condition of the internal organs, forced alimentation should be regarded, not as a means of affording nourishment, but of administering suitable medicines.

III. *History of an extraordinary case of Nervous Disorder terminating fatally.* By Dr. Meugy, *fils.*

This case, which is, indeed, extraordinary and full of interest, was of so long duration, and so various in its phases, that its narration extends over sixteen pages of the journal.

A young lady, at the age of thirteen years, was of moderate growth, delicate constitution, but good general health. At the age of eleven years she suffered from cerebral symptoms, after the sudden disappearance of an eruption of scarlatina. Palpitation of the heart and severe headaches were experienced from time to time. At the age of fourteen the headaches greatly increased in severity, with an intolerance of light, and sound, and movement, and were frequently accompanied with epistaxis. The pulsations of the carotid arteries were violent and perceptible. Compression of these arteries not only arrested the painful violence of their pulsations, but also relieved other symptoms, and health was completely re-established for a whole year, during which menstruation occurred regularly.

At the end of the year all the symptoms returned, with aggravation—pressure on the carotids only increased their severity. Medicines were of no avail. The patient became sleepless, and now, occasionally, choreic movements of the limbs were noticed. Food was refused, or scarcely any taken. The bowels costive; urine scanty and sedimentary. These symptoms continued for four weeks, when suddenly the patient fell into a sound sleep, which lasted one night, and in the morning her health seemed entirely restored.

A short time afterwards, she began to suffer from toothache, which persisted, despite all the skill of the dentist; and soon to this was added again intense headache, which compelled her to keep her bed. This headache was more severe than ever, and was accompanied with tremors of the muscles of the neck, by which the head was constantly jerked backwards; violent spasmodic flexion of the legs and thighs also took place. She complained constantly of the intensity of the pain in the head. She slept only for about an hour towards morning. Food and beverage were obstinately refused. · The pulse 80 or 90, and rather feeble; the action of the heart calm, although the patient complained of the feeling of insupportable pulsation. The patient was becoming emaciated. The intellectual faculties remained unimpaired. The patient desired to be kept in constant darkness. She exclaimed constantly, "Hold my head." Medicine only aggravated the symptoms. This state continued for a month, the symptoms being rather increased in severity, when one night she fell asleep, and, as before, woke on the following morning perfectly well.

This restoration to health lasted only three weeks. The symptoms again appeared, with the addition of more violent convulsive movements. The limbs were thrown into a succession of most rapid movements. She would strike the bed with her heels ten, twenty, or fifty times, and then sink exhausted, to repeat the same movements after an interval of a few minutes. It was utterly impossible to restrain these movements. Tetanic rigidity of the arms lasting four weeks, followed the immersion of the hands in warm water; any attempt to bend them caused shrieks of agony. Again these symptoms all suddenly disappeared after a night's sleep.

In about three weeks, another attack somewhat similar to the previous occurred, with the exception that, suddenly, the patient, lying on her side, began to beat the pillow with her head, as many as from twenty to fifty blows at each paroxysm. At the last and strongest blow, she would sink down, crying, "Oh my head!—hold my head!" These paroxysms were repeated every five minutes without interruption during three weeks, except when rarely interrupted by an hour's sleep. Retention of urine rendered catheterism necessary for two days. At the end of three weeks, a more violent and lasting paroxysm occurred; she beat her head on the pillow at least a hundred times, and then suddenly feel asleep for nearly twenty-four hours, and woke with re-established health.

The restored health lasted a long time; the patient undertook a journey to Dieppe, and returned still in good health. It must be observed that menstruation had continued to appear regularly throughout the attacks.

In about three months' time, after having attended a religious ceremony, which appeared at first to have exercised a most beneficial effect over certain premonitory symptoms which had showed themselves, she fell into a state of languor and suffering as before. At this time, electricity, by the machine, was administered at the patient's desire, but without any benefit. In a few weeks, she became much worse, laid constantly on one side, her head buried in the pillow, refusing to see the light or take any kind of nourishment, complaining of headache and toothache, remaining with her legs flexed on her thighs, and the thighs on the pelvis. Occasional twitching and starting of the limbs were noticed, which Dr. Meugy compares to electric discharges. Intermittent fever supervened, over which quinine exerted no influence, and the ague became changed to continued fever, which, however, subsided of itself in a few days. She remained in the same state to the expiration of four months from the beginning of the paroxysm. One day she suddenly straightened herself on her bed like a spring forcibly unbending itself. She then leaped from her bed, and jumped about the room for a few minutes; then returned to her bed; again she leapt about the room, and again she returned to bed exhausted. Then she sprang from her bed and ran round the room, stopping in one corner, where she danced many times in succession on her heels. If she met any one when running round the room, she would avoid the person, and shriek out that a wild beast was in pursuit of her. Her eyes were fixed, haggard, and dull. At last she would fall exhausted into the arms of her father or mother, exclaiming, "Oh my head!"

During three weeks, this irresistible impulse to run would return three or four times in an hour, and any attempt to restrain her produced convulsions and agonizing shrieks. No febrile symptoms were observed; the pulse remained regular but feeble, the skin of its natural temperature. On one occasion, after a paroxysm longer and more severe than usual, the nervous symptoms assumed another form. The patient would kneel by her bedside, and bend her head from side to side like the oscillations of a pendulum; then, after a few minutes, resume her bed, exclaiming, "Oh my head!" Immediately afterwards, rapid successive cracking of the joints of the fingers would be heard, resembling slight electrical discharges; they were excited by movements of flexion and circumduction, which she unceasingly performed. These were occasionally interrupted by a deep sigh and a few minutes' rest. She continued in this state for several weeks, when these symptoms unexpectedly subsided, to give place to other phenomena. The patient, lying on her back, would suddenly rise into the sitting posture, then forcibly throw herself back on to her pillow. She repeated this movement thirty or forty, or even a hundred times in succession. It was necessary that these movements should be aided by some person, otherwise a tetanic rigidity of the whole trunk ensued, with piercing cries, which continued until the former movements were resumed. In the brief intervals which occurred, the cracking noises before-mentioned were repeated. This state also lasted for several weeks, and then passed off in a greatly augmented paroxysm, followed by sleep.

The general health improved, some face-ache which remained was relieved by the extraction of several carious teeth, after which the patient rapidly, and apparently completely recovered.

In about six months' time, her parents took her from home for the benefit of change of air and scene. Dr. Meugy warned them, that as medicine had on every occasion not only failed to relieve, but had, moreover, rather served to aggravate the symptoms, they should on no account, in the event of the symptoms returning, suffer her to be actively treated. This advice was not followed. Soon afterwards another attack occurring, at Rheims, she was placed under medical care, and treated for congestion of the brain. The paroxysms now acquired such intensity that she died, with acute fever, laryngitis, and ophthalmia, superadded to the other symptoms which had continued during seventeen months.

Dr. Meugy considers that the only explanation which can be offered of this extraordinary case is to suppose a disturbed condition of the electricity of the body, and regrets that accurate observations were not made on this point; and further, he inclines to the opinion that as each paroxysm was terminated in sleep, mesmerism would have arrested and controlled the attacks had it been practised.

We have here presented our readers with a very full abstract of this truly unparalleled case, but must add that the original memoir will well repay its study.

IV. *A Medico-legal Report on a Case of Imbecility, complicated with Melancholy. Violation of Young Girls.* Reported by M. Girard, of Auxerre.

This case contains the disgusting details of the acts of a poor idiot youth, in whom erotic feelings were dominant, and impelled him to the violation of female children. M. Girard remarks, "It is well known that *salacity* is one of the attributes of idiocy; that the unfortunate idiot, left to his own solitary thoughts, incapable of selecting associates of his own age, selects those who, in the feebleness and timidity of infancy, approach to the condition of his own faculties;" and so this poor lad, with strong sexual instincts, gratified them where he encountered the least resistance.

V. *On the Clinical Study of Mental Diseases.* By M. Falret, of the Salpetrière.

The subject of this paper forms the continuation of one published in the preceding number of the journal.

M. Falret observes that the clinical study of mental diseases has been objected to on two grounds—1st, the disadvantages likely to accrue from the introduction of strangers into the asylums; 2nd, the supposed evils attendant on the public examination and interrogation of the insane.

M. Falret proceeds to combat these objections, and to show that the attendance of visitors as students, under the superintendence of the physician, will tend to produce increased vigilance, and more careful performance of duty by all the subordinate officers of the establishments for the insane. That so far from being injurious to the insane, it operates beneficially by drawing them out of themselves, and the contemplation of their own thoughts and feelings. M. Falret remarks that a broad distinction exists between the visits of those attracted to an asylum through mere idle curiosity, and the grave and earnest occupation of students of medicine.

In referring to the interrogation of the patients, M. Falret points out that the fears which are entertained on this score are not well founded, because they are drawn rather from the feelings of the man of sound mind than from the insane. M. Falret gives three general characters by which the insane is to be distinguished from the sane man. First, the insane is in a false position with regard to other men, sees through a false medium, and with false perceptions, the whole external world. Second, the concentration of the patient's thoughts upon his own internal world, constituting an entirely internal life. Third, this internal life in which the insane is absorbed, breaks his tie with his fellow-man.

In this point of view, M. Falret arranges the insane in three groups. The first are impelled either by the pleasure which they experience in making themselves prominent objects, or in speaking of their own ideas, or in complaining of the indignities and persecutions to which they are subjected, and so delight to obtrude themselves on the notice of others. The second, although occupied with their own reflections, and their thoughts concentrated upon the objects of their delirium, are not averse to making these known when solicited thereto. The third are too much afflicted, or are too weak in intellect, to be wounded by any questions that are addressed to them, although not quite ignorant of what is taking place around them.

M. Falret argues at considerable length that in the several forms of dementia, mania, and partial insanity, to each of which forms of mental disease in relation to clinical instruction he devotes a separate consideration, the influence of examination and interrogation before the students is calculated to produce a beneficial, instead of an injurious influence upon the patients. The author readily disposes also of several indirect objections to the clinical study of mental disease; *e. g.*, the revelation of family secrets, the loss of time to the attendant physician, the imperfect knowledge of mental disease afforded thereby, the increase in the number of asylums it will demand, and lastly, the absolute right to institute such a *clinique.*

M. Falret advocates as the best mode of instruction, the taking the students round the establishments, in preference to the introduction of the patient into the clinical theatre. The patient is thereby seen in his real state, and the order of the institution is not interrupted. The rules which should guide the conduct of the students and of the instructor are also carefully laid down by M. Falret; but as there exists little difference of opinion in England as to the importance of clinical study of mental disease, there is little occasion to occupy further our readers' attention with this very excellent essay.

### VI. *Reviews of Medical Journals.*

M. Piorry relates (in the *Gazette des Hôpitaux*) a series of cases of insanity, in which the administration of the sulphate of quinine appears to have been attended with success.

*Case of Mania determined by Lumbar Abscess.*—This was an instance of violent mania, induced immediately by recent political events in France, all the symptoms of which rapidly and permanently disappeared after the opening of a large lumbar abscess.

*The Revue Médicale* also supplies several cases, from among which we notice *a case of hysteria complicated with amenorrhœa and hæmoptysis, and abstinence lasting nineteen months*, by M. Ferrand de Missol. The patient was eighteen years of age. Amenorrhœa preceded the hysteria; the hysterical attacks assumed the severest or epileptic form. During six months previously to admission into the asylum, the patient constantly vomited everything she took into her stomach. For fifty-three days after she came under the care of M. Ferrand de Missol, she only moistened her mouth with water. During the whole of this period she had only two alvine evacuations; during the remainder of the period she continued to reject food. She ultimately recovered on the appearance of an eruption of acne. One peculiar feature was observed in this case, that pressure on the epigastrium arrested the convulsions. The eruption of acne appears in this case to have been critical.

*Pregnancy complicated with ecclampsia,* requiring the induction of premature labour, and followed by puerperal mania, is extracted from the *Archives de Médecine Belge.* The pregnancy was at the eighth month; the convulsions were attended with unconsciousness, a comatose state, with dilated pupils. Premature labour was induced; the patient progressed favourably. On the second day afterwards, puerperal mania appeared; this subsided in two days under the use of sedatives.

*A case of iodic intoxication* is copied from the *Archives de Médecine Belge,* in which all the symptoms of drunkenness appeared in a man after prolonged use of iodide of potassium. The symptoms, which resembled those of poisoning from lead, lasted for four months, and ended in insanity, in a paroxysm of which he destroyed himself by throwing himself from a window.

M. Delasiauve relates a case of *organic lesions found in a case of epilepsy,* and which consisted of two osseous concretions attached to the dura mater, one projecting into the anterior, the other into the posterior lobe of the hemispheres.

*On General Paralysis.* By M. Belhomme. — The author laying down the conclusion that general paralysis is always the result of organic disease of the brain, and that loss or impairment of speech is always caused by disease of the anterior lobes.

*On the Signs of Contusion of the Brain,* by M. Boinet, who draws an analogy between this lesion and ramollissement of the brain.

The last section of the *Annales Medico-Psychologiques* comprises bibliographical notices of the following publications: "On the Climate of Italy," by Dr. Carrière; "The Physicians' Report of the Royal Hospital of Bethlehem;" "The Treatment of Insanity," by Dr. Billod; "Medico-legal Considerations on Insanity," by Dr. Dagouet; "Clinical Observations on the Localization of the Cerebral Functions, and on Insanity," by Dr. Belhomme.

